# The international treaty for pandemic preparedness and response: same story, different times?

**DOI:** 10.2217/fvl-2021-0214

**Published:** 2021-10-28

**Authors:** Dominique Vervoort, Xiya Ma, Alia Sunderji, Hloni Bookholane

**Affiliations:** 1 ^1^Institute of Health Policy, Management & Evaluation, University of Toronto, Toronto, ON, M5T 3M6, Canada; 2 ^2^Division of Plastic Surgery, Faculty of Medicine, Universite de Montreal, Montreal, QC, H3T 1J4, Canada; 3 ^3^Department of Emergency Medicine, The Hospital for Sick Children, Toronto, ON, M5G 1X8, Canada; 4 ^4^Department of Medicine, University of Cape Town, Cape Town, South Africa

**Keywords:** coronavirus, COVID-19, global health, health policy, pandemic, preparedness, prevention

## Abstract

In November, dozens of nations and the WHO will draft the international treaty for pandemic preparedness and response. Will the treaty be the needed change in global health equity or are we doomed to repeat history?

The narratives of infectious disease outbreaks have long been dominated by inequities. COVID-19 has significantly affected minoritized and disenfranchised populations in high-income countries and is overwhelming health systems in low- and middle-income countries (LMICs). The pandemic has cost the lives of millions of people in mere months [1]; as a result, the pandemic represents the largest socioeconomic and health crisis in modern history since World War II. Simultaneously, there remain overlooked pandemics, such as TB and HIV/AIDS, which precede the COVID-19 pandemic. Every 22 s, someone dies from TB; whereas, 38 million people are currently living with HIV/AIDS [2]. The relative containment of these infectious diseases in high-income countries has resulted in limited media attention, research and funding to mitigate or eradicate these conditions in LMICs. COVID-19 may face a similar fate unless concrete actions are taken.

Now, high-income country actors have agreed on developing an international treaty on pandemic preparedness and response to mitigate future crises. But, are we doomed to repeat history and cater only to the rich nations?

## Global health inequities

Disparities in healthcare are obvious and omnipresent worldwide. A minority of nations currently provide universal health coverage to their populations [3]. Life expectancy at birth ranges from 53 years in the Central African Republic to 85 years in Hong Kong [4]. Five billion people lack access to safe, timely and affordable surgical, obstetric and anesthesia care when needed, causing over 17 million preventable deaths each year [5]. Without investments in healthcare infrastructure and delivery, the world’s poorest countries, regions and populations will drift further away from wealthier ones.

These disparities, as pervasive and perverse as they already were, have been exacerbated by the COVID-19 pandemic. The pandemic has served as another litmus test for global health equity in the modern era; a test the world failed, yet again. High-income countries rapidly hoarded equipment (e.g., ventilators), disposables (e.g., personal protective equipment) and vaccines, leaving LMICs far behind [6]. As LMICs struggle to get a small percentage of their population vaccinated, countries like Canada pride themselves in having ordered five-times as many doses as needed [7], moving on to offer third optional doses to citizens seeking to travel abroad and seeing doses needlessly expire [8,9]. Further, high-income countries, especially in Europe, engaged in geopolitical fights, which included contentious vaccine distribution changes in the early aftermath of the UK’s Brexit and the halt of exports of COVID-19 vaccines out of Europe. Moreover, high-income countries proceeded to block attempts of LMICs to waive vaccine patents under the World Trade Organization. As a result, LMICs are not expected to see their populations be fully vaccinated and protected against COVID-19 until 2024 [10]. Baseline healthcare services in LMICs, which were already scarce, have become even less available as countries burn through their personal protective equipment, critical care capacity and other resources [11]. As these resources shift toward meeting the needs of COVID-19 patients, funds and attention toward other healthcare services have shrunk, brewing a future crisis that will last for years to come [11].

It has become apparent that there is a need to recalibrate global health, what it means and what it represents [12]. Global health, in its current state, often serves as a means to put one’s own political and economic agendas first, defeating its true purpose of equity and unification [13]. Similar to the HIV/AIDS and TB pandemics, politically and economically powerful nations rushed to help themselves first, often at the cost of other nations. This resulted in a subsequent savior mentality of ‘helping’ exactly those countries which have been the victim of their initial, self-serving actions. And yet, global health inequity affects all of us. The ripple effects of a global sanitary crisis like the current pandemic and broader crises such as climate change illustrate that no one is safe until everyone is safe. The neglect of LMIC-based disparities will ultimately affect other countries worldwide.

## International treaty on pandemic preparedness & response

In response to the severe COVID-19 repercussions in Europe and the glaring gaps in the global pandemic response, the European Council President, Mr Charles Michel, called for the development of a global treaty in December 2020 to put an end to this once-in-a-century crisis. The proposed international treaty for pandemic preparedness and response was approved by 26 Heads of State and the WHO Director-General, Tedros Adhanom Ghebreyesus, on 30 March 2021 [13]. However, initial letter signatories did not include the geopolitical powerhouses that are the USA, China and Russia [14]. These countries are three of the five permanent UN Security Council members and major suppliers of vaccines, personal protective equipment and other essential equipment. The pandemic is not only a global health but also a health security issue, necessitating commitments by all.

In May 2021, the virtual World Health Assembly, themed ‘Ending this pandemic, preventing the next one,’ generated support from all individual EU members and over 30 countries, including the USA but still excluding China and Russia, to draft the treaty in November 2021 [15]. Only select non-European LMICs were included in or supported the draft decision, being Ecuador, Guyana, Libya, Paraguay, the Philippines and Sudan, requiring efforts for increased inclusion at the time of development. Although the WHO itself is underfunded and limited in legislative power and implementation, such a treaty may unite countries more effectively and result in improved accountability. It has done so for the WHO’s Framework Convention on Tobacco Control, the sole time in history where the WHO used its treaty powers but which resulted in unprecedented international health cooperation [16]. But, will the treaty share the same positive fate?

## Promises & realities

In anticipation of the treaty, the European Commission outlined ten key incentives underlying the need for a global treaty [17]. The incentives are encouraging and serve as a hopeful roadmap toward greater global health equity in pandemic preparedness and responses. Realistically, a deeper dive will be necessary.

The treaty promises to bring ‘greater certainty for citizens regarding equitable access to pandemic countermeasures’ and ‘fair accountability systems’. The latter is critical, as accountability mechanisms to date have been limited, flawed or not reinforced. Countries’ willingness to develop a more equitable treaty is laudable; however, sanctions need to be introduced and reinforced if signatories do not comply with the treaty’s promises. Moreover, ‘a guaranteed seat at the table’ is promised for all. The World Health Assembly and UN General Assembly, while excluding select nations and territories, have ensured relatively inclusive seats at the table. Yet, despite having a voice, the prioritization of health needs has often been driven by funders rather than community needs whereas implementation efforts in LMICs have been insufficient. At last, countries’ core capacities are to be clarified; the baseline capacity to respond to healthcare crises such as the COVID-19 pandemic has been variable between and within countries, with some relying on a single ventilator or barely an intensive care bed per million people [18].

It becomes clear that specific and concrete promises must be made to ensure implementation, monitoring, evaluation and accountability. Whether such measures can be reinforced and independently regulated remains unclear, especially so given the pervasive geopolitical nature of today’s world, but a prerequisite to establishing such a treaty.

## Opportunities for moving forward

*Duff* *et al.* [19] have proposed a WHO-like global body that can coordinate governments, set up transnational operations, enforce treaties and rules through incentives and penalties and provide objective, clear and scientifically sound technical advice to prepare for and respond to infectious disease outbreaks. Such an entity would require full political autonomy and sustained and sufficient funding for its operations, two characteristics that seem wholly unrealistic in light of modern geopolitics. Conversely, in their open letter to the UN, *Chiriboga* *et al.* [20] have called for the establishment of a multisectoral Global Health Equity Task Force within the WHO to ensure equitable pandemic responses and global health delivery mechanisms. While this would not address the limitations faced by the WHO, including funding constraints and members geopolitical weight, it may serve as a realistic stepping stone for further developments. Undoubtedly, there is an urgent need to re-establish trust between nations and toward the WHO, especially in the eyes of the public. Though unable to do so in a legally binding manner, commitments to WHO resolutions and WHO-driven or other international treaties must be recognized by nations as binding, even if virtually, in a similar manner as the WHO Framework Convention on Tobacco Control.

Ultimately, reality may be a roadmap toward these ideal values. The attainment of all promises laid out by treaty constituents and stakeholders seems opportunistic, almost utopic. However, a foundation needs to be laid to build toward a more equitable future. Heads of state, international organizations and other stakeholders are at a crossroads: will the same old story be retold or will the COVID-19 pandemic be an opportunity to acknowledge the mistakes from the past and reassess the health disparities faced throughout the world?
